# Healthcare Personnel Interactions with Floors and Pathogen Transmission in Long-Term Care: A Qualitative Exploration

**DOI:** 10.1017/ash.2024.261

**Published:** 2024-09-16

**Authors:** Emily Chasco, Kimberly Dukes, Loreen Herwaldt, Michaela Zimmer

**Affiliations:** Institute for Clinical and Translational Science, University of Iowa / Iowa City VA Health Care System; Dept of Gen Int Med, Carver College of Medicine, University of Iowa; University of Iowa Carver College of Medicine

## Abstract

**Background:** We know relatively little about how healthcare personnel (HCP) in long-term care facilities (LTCFs) integrate hand hygiene (HH) and personal protective equipment (PPE; e.g., gloves) use into their care processes. To address this gap, we are examining HH and PPE adherence at critical and contaminating moments in LTCFs. **Methods:** We conducted ethnographic observations of HCP’s processes of care in 2 LTCFs in Iowa to examine HH and PPE adherence during contaminating tasks in resident care sequences. We captured care observations and additional data on topics related to our study focus (e.g., unit/room layouts, PPE storage, facility policies/procedures) in field notes. We transcribed and imported fieldnotes into MAXQDA qualitative data management software and analyzed the data using a combined deductive-inductive coding approach. **Results:** Between 1/2023-7/2023, we observed 60 (30 per facility) care episodes. Most observations included toileting activities and perineal care during which HCP would be expected to use gloves and/or do HH. Most HCP appropriately donned/doffed gloves and practiced HH at key moments (e.g., before clean/aseptic procedures, after perineal care), but were less compliant before/after touching residents’ clothing or bare skin during these activities. In addition, some held soiled items next to their scrubs between tasks, which could contaminate their clothing and arms and could facilitate transmission of pathogens to other residents. Moreover, HCP’s interactions with floors emerged inductively as a topic of interest during our observations and preliminary analyses. We observed HCP interact with the floor during these activities in ways that could increase risk of pathogen transmission. HCP frequently dropped soiled towels or wipes used in perineal care onto the floor during tasks for later pick up. HCP also moved or placed trash bags containing soiled or contaminated items on the floor. HCP routinely knelt on, sat on, or touched their hands on the same floor when talking with residents, helping residents change clothes or diapers, changing bedding, or adjusting wheelchair footpads. In one case, the HCP picked up clean towels that fell to the floor near soiled towels and then used the “clean towels” in resident perineal care. **Conclusion:** Despite practicing HH and appropriate PPE use, HCP in LTCFs may increase the risk of pathogen transmission unintentionally through their interactions with soiled items and the environment, including floors. Given the nature of resident care in LTCFs, HCP in LTCF may be more likely than HCP in acute care settings to interact with contaminated floors.

